# How is Clinical Trial Reimbursement Money Spent? South African Trial Participants’ Reported Reimbursement Spending Patterns and Perceptions of Appropriate Reimbursement Amounts

**DOI:** 10.1007/s10461-021-03418-2

**Published:** 2021-08-11

**Authors:** Cecilia Milford, Tammany Cavanagh, Yolandie Ralfe, Virginia Maphumulo, Mags Beksinska, Jennifer Smit

**Affiliations:** MRU (MatCH Research Unit), Department of Obstetrics and Gynaecology, Faculty of Health Sciences, University of the Witwatersrand, 40 Dr AB Xuma Street, Commercial City Building, Durban, 4001 South Africa

**Keywords:** Reimbursement, Clinical trial participation, South Africa, Vulnerable participants

## Abstract

Reimbursement of participants in clinical trials is extensively debated. Guidance recommends that compensation should reflect time, inconvenience and reimbursement of expenses. This study describes how participants spend their reimbursement and perceptions of appropriate reimbursement amounts. This was a sub-study of the evidence for contraceptive options and HIV outcomes (ECHO) trial. Participants were from two sites in KwaZulu-Natal, South Africa. A mixed methods approach was used. 500 participants completed a questionnaire, and 32 participated in one of four focus group discussions (FGD). The majority (81%) used reimbursement for transport to the research site, followed by toiletry purchases (64%). Many described how reimbursement supplemented income, used to cover basic living costs. Some used money to buy luxury items and takeaway foods. The ideal reimbursement amount per visit ranged: ZAR150-ZAR340 (US$10–24). Reimbursement spending and perceptions are in line with local guidance. Reimbursement should consider risk minimization together with ensuring informed, voluntary decision making.

## Background

The purpose of reimbursement in research and clinical trials is to compensate participants for their inconvenience and time as well as any expenses incurred, such as transport costs [1–6]. Although guidelines exist for reimbursement of trial participants [5, 7–12], acceptable reimbursement rates are under researched. There is also a paucity of literature on how participants actually spend their reimbursement money.

Reimbursement of participants in research and clinical trials is an extensively debated issue worldwide [3, 4, 6, 13–16]. There have been concerns that the practice of paying research participants could induce people to participate in a study they would not otherwise take part in [15, 17]. Substantial reimbursement offers may be especially enticing to vulnerable groups who may disregard risks to their safety for the financial gain [18–20]. It has also been argued, however, that participants are entitled to appropriate compensation for their personal contribution to scientific advances [21], and that poor/vulnerable communities should be remunerated as long as study-related risks are fully minimized [19, 22]. Empirical research has demonstrated that monetary compensation does not appear to lead to participants ignoring possible study-related risks [15]. In general, local norms and standards should be considered when determining compensation and reimbursement rates [3].

In South Africa, the question of appropriate reimbursement of participants also remains contentious. Drawing on international debate, the National Health Research Ethics Council (NHREC) [21], note that, given the socio-economic situation of many South Africans, a large proportion of the population is considered vulnerable, and that even minimal payments may provide unfair incentives and reduce proper risk consideration [21]. On the other hand, they state that participants deserve appropriate compensation for their contribution to clinical trials [21]. Therefore, they published guidelines for consideration by research ethics committees/institutional review boards (RECs/IRBs) and researchers on the matter of reimbursement payments. They recommend differentiated payments between studies according to the research burden imposed on participants, and which reflect compensation for time, inconvenience, and reimbursement of expenses: the TIE (time, inconvenience, expenses) compensation model for payment of research participants [21]. In 2018, the South African Health Products Regulatory Authority (SAHPRA) issued a model to advise calculations using the TIE compensation guidelines and recommended that a minimum payment of ZAR300 (South African Rands) (equivalent to approximately $20.60)^1^ be paid per standard participation visit in a clinical trial [7].

The South African economy has high levels of unemployment, with 29.1% of the population unemployed in 2019 [23], the highest proportion of which were black Africans (33.8% in first quarter 2020) [24]. More South African females than males, aged 15–64 years, were unemployed in the first quarter of 2020 (32.4 and 28.3% respectively)[24]. There are various social grants available to support South African citizens, including old age pension, child support, disability and foster care grants [25].

Research on reimbursement of participants in South Africa is limited. How reimbursement money is spent by study participants is not fully understood or documented. There is also a need to further explore what amount is not considered excessive and that does not constitute undue inducement, yet at the same time protects against exploitation of vulnerable communities [1], especially in the poor economic context of South Africa. This sub-study provides descriptive data on how reimbursement money is allocated for spending by trial participants at two study sites in South Africa, and their perceptions of appropriate and acceptable reimbursement amounts.

## Methodology

This research was a sub-study of the multi-center, open-label, randomized clinical trial, comparing HIV incidence and contraceptive benefits in women using depot medroxyprogesterone acetate (DMPA-IM), levonorgestrel (LNG) implant, and copper intrauterine devices (IUDs), the evidence for contraceptive options and HIV outcomes (ECHO) trial [26]. The ECHO trial was conducted in 12 sites in four countries—South Africa, Kenya, Zambia and Eswatini. Women aged 16–35 years were invited to enroll into the ECHO trial if they desired effective contraception and were willing to be randomised to any one of the three trial contraceptive methods approved for use in South Africa (DMPA-IM, LNG or IUD). Follow-up visits occurred at one month, two months, and every three months thereafter, up to 18 months [26].

This sub-study was conducted in 2018 at two of the ECHO trial sites in KwaZulu-Natal (KZN), South Africa, in Umgungundlovu (Pietermaritzburg) and eThekwini (Durban) Districts. The site in Umgungundlovu District was located in a peri-urban, formal township area. Participants attending this site came from both peri-urban township and more rural locations around the study site. The site in the eThekwini District was located in the Durban city centre. Participants attending this site resided in a range of locations around and within the city.

This sub-study had a mixed methods approach. A quantitative (interviewer-administered) questionnaire was conducted with 250 ECHO study participants returning for their month 15 visit at each research site (500 participants in total). In addition, two focus group discussions (FGDs) were conducted per site (four FGDs in total), with groups of women who were attending retention study visits at the respective research sites. FGD participants did not complete the quantitative questionnaire at the time of the FGD. They may or may not have completed the questionnaire at their month 15 visit, but there was no link between data or participation in the two study components, rather the two data sources were used for data triangulation. Both quantitative questions and qualitative discussions explored what participants actually spent their reimbursement money on, and their perceptions of appropriate reimbursement amounts.

During the main ECHO trial, participants were reimbursed ZAR150 (approximately US$10) and were provided a meal, at each clinical study visit at these two study sites. Interim study visits (generally to attend to method related side effects or method related problems, e.g. method expulsion) were reimbursed with ZAR100 (just less than US$7). These reimbursement amounts were approved by ethics committees in 2015, prior to the revised 2018 SAHPRA TIE compensation guidelines [7]. Participants in this sub-study were not provided with any additional reimbursement for their participation in the sub-study, as they were already receiving reimbursement and a meal for attending their clinical trial follow-up visit (ZAR150, ~ US$10) or retention study visit (ZAR50, ~ US$3.50).

### Data Analysis

The quantitative data were entered onto a REDCap database [27] and were descriptively analysed. The qualitative data were transcribed and translated from *isiZulu* into English. Qualitative data were coded using deductive and inductive codes and were thematically analysed. NVivo version 11 (QSR International) was used to facilitate data analysis. The different data sets were used for data triangulation, and to check the reliability of the findings.

### Ethical Considerations

This was a sub-study of the ECHO clinical trial [26], and was approved by the University of the Witwatersrand’s Human Research Ethics Committee (HREC) (Ref: 141112) in October 2017. All participants provided written informed consent. Separate consent was obtained for the quantitative and qualitative components, and participants in the FGDs also provided written consent to the audio recording of the discussions.

## Results

### Participant Socio-Demographics

A total of 500 female participants completed the quantitative questionnaire (250 from eThekwini and 250 from Umgungundlovu District), and 32 participated in the FGDs (16 at each research site).

Participants in the quantitative component were aged between 19 and 36 years, with a mean age of 24.4 and 24.5 years in the eThekwini and Umgungundlovu sites, respectively (Table 1). At both sites, participants had a range of 0–4 children. Overall, one-third of participants in the quantitative component lived more than 20 km from the study site. Over half (56.8%) of participants from eThekwini, the urban centre, lived more than 20 km from the study site, whereas in Umgungundlovu, half (50.4%) of participants lived within 5 km from the study site. FGD participants were not asked for information on their age, number of children, or distance of residence from the study site.

**Table 1:** Basic demographics of participants who completed quantitative questionnaire

Participant background demographics details	eThekwini	Umgungundlovu	Total
Number of participants (quantitative questionnaire) (n)	250	250	500
Age in years (mean, range)	24.4 (19–34)	24.5 (19–36)	24.4 (19–36)
Number of children/parity (mean, range)	1.2 (0–4)	1.1 (0–4)	1.1 (0–4)
Distance participants live from research sites % (n)			
0–5 km	2.4 (6)	50.4 (126)	26.4 (132)
6–10 km	5.2 (13)	27.2 (68)	16.2 (81)
11–20 km	35.6 (89)	11.2 (28)	23.4 (117)
> 20 km	56.8 (142)	11.2 (28)	34.0 (170)
Monthly income (ZAR): range	R0–R10000	R0–R12000	R0–R12000
Mean	R1550.85	R1572.68	R1561.81
Median	R1000	R1000	R1000
Source of financial support % (n)			
Social grants^a^	64.8 (162)	65.2 (163)	65.0 (325)
Male partner	39.6 (99)	44.8 (112)	42.2 (211)
Other family/remittances	31.6 (79)	31.6 (79)	31.6 (158)
Full time formal employment	14.4 (36)	16.4 (41)	15.4 (77)
Part time formal employment	14.8 (37)	10.8 (27)	12.8 (64)
Self employed	4.8 (12)	6.0 (15)	5.4 (27)
Casual work/day labour	3.6 (9)	7.2 (18)	5.4 (27)
Other	0 (0)	2.8 (7)	1.4 (7)
Transport costs to the research site (ZAR)^b, c^			
Range	R0–R540	R0–R500	R0–R540
Mean	R44.74	R27.77	R36.36
Median	R30.00	R20.00	R26.00
Ever had to borrow money to cover travel expense for study visits % (n)			
No, have never borrowed money	38.8 (97)	49.6 (124)	44.2 (221)
Yes, have borrowed money	60 (150)	43.6 (109)	51.8 (259)
Do not pay transport expenses to get to research site	1.2 (3)	6.8 (17)	4.0 (20)
Borrowed transport money from^d^			
Female family member	52.7 (79)	34.9 (38)	45.2 (117)
Friend	38.7 (58)	37.6 (41)	38.2 (99)
Male family member	0.7 (1)	6.4 (7)	3.1 (8)
Boyfriend	3.3(5)	0.9 (1)	2.3 (6)
Neighbour	12.7 (19)	20.2 (22)	15.8 (41)
Other	3.3 (5)	0	1.9 (5)

^a^Actual type of social grant was not explored. The monthly value of these grants depends on type of grant (at the time of the study (2018), pensioner and disability grants were approximately R1690 (~ US$ 116.50), child support grants were R400 per child (~ US$27.50), and R920 (~ US$63.50) for foster care [28]

^b^At an exchange rate of approximately US$1 to ZAR14.50 (February 2021)

^c^Some participants relocated during the course of the study resulting in increased transport costs, therefore the upper range of transport costs is high for both sites, and mean is higher than median

^d^Some participants provided multiple responses

Participants in the quantitative component reported a range in monthly income from zero income to ZAR12000 (~ US$827), with a median of ZAR1000 (~ US$69) per month across both sites (Table 1). Their source of monthly financial support was predominantly from social grants (65.0%) and male partners (42.2%), and some participants reported multiple sources of financial support. Very few participants had full time employment (15.4%). Of the seven participants that listed other income sources, six had no income [28].

Although FGD participants did not discuss monthly income, they described their source of financial support/income. Similar to the questionnaire data, social grants were described as the most common source of financial support by FGD participants. Many FGD participants reported living with their maternal families in large households supported by few, if any, wage earners. Most FGD participants described that they were unemployed and relied on a combination of income sources including grant money, support from others (including male partners and other family members), and/or part-time jobs or “piece” work (referring to casual/day labour). Some FGD participants also discussed the study reimbursement as complimentary to their income.

*[W]e are eleven in my family. The money comes in but mainly what comes in is this children’s grant because there are other two sisters of mine who get child grant. And I also get the children’s grant. And my husband sometimes works, although he doesn’t work in something stable but with that income we are able to put things together with it, and be able to sustain ourselves, although it does not satisfy us but we are able to live. (Participant 7, eThekwini, FGD 2)*

*I’m unemployed and eh I live with my family. My mom is a breadwinner at home. […] I have one son. Eh my income is grant and also my mom keeps, supports the family. Ehm, and I think my income, when I add the money that I get here [study reimbursement], it makes a lot of things, but I’m not yet satisfied [with total income]. (Participant 3, Umgungundlovu, FGD 2)*

Participants who completed the quantitative questionnaire were asked how much they spent on transport to and from the research site. Participants from eThekwini on average spent slightly more on transport to their research site per visit than those from Umgungundlovu [median expenditure: ZAR30 (~ US$2) versus ZAR20 (~ US$1)] (Table 1). Some participants relocated during the course of the study, and subsequently their transport costs increased dramatically—therefore the upper range of transport costs is high for both sites [ZAR500 (~ US$34) and ZAR540 (~ US$37) respectively]. Twenty participants paid ZAR100 or more (> US$6.50) for their transport costs (13 from eThekwini, and 7 from Umgungundlovu). Participants whose transport cost ZAR100 or more (> US$6.50), due to relocation, were able to ask for additional reimbursement (although not all did ask for more—and reasons for this were not explored). With ethics approval, these participants were reimbursed to cover their increased transport costs.

More than half (51.8%) of participants in the quantitative component (Table 1) also noted that they had borrowed money, at some point, to get to the research site. The most common sources of borrowing money were from a female family member (45.2%), a friend of undisclosed gender (38.2%), or a neighbour (15.8%). A small proportion reportedly did not pay transport fees to get to the study site (4.0%), mostly because they were locally based and could walk to the study site.

Although FGD participants were not asked directly if they had borrowed money to travel to the research site, a few mentioned they had used their reimbursement money to pay back money they had borrowed for transport (not specifying if transport to the research site or if transport in general).

*I […] spend it [reimbursement] on transport. If I had borrowed it I pay it back again. It also helps me when I travel to school, if maybe my brother did not give me [money] on that day.* *(Participant 3, eThekwini, FGD 1)*

### Reimbursement Spending Patterns

Participants from the quantitative component specified how they most often spent their reimbursement money (Table 2)—multiple responses were allowed for each participant. Although there were variations in what money was spent on, the majority (81.0%) reportedly used money for transport to the research site. This was followed by purchase of toiletries (64.0%). A small portion from each research site used money for childcare whilst they were at their study visits (although participants were welcome to bring their children to the study sites). There were slight variations in spending patterns across sites, as the quantitative data indicate that more Umgungundlovu participants spent money on groceries, clothes, hair care, and takeaways, than eThekwini participants. Slightly fewer participants from Umgungundlovu spent money on transport compared to eThekwini participants, and 77.6% of Umgungundlovu participants lived within 10 km of the research site, compared to 7.6% of eThekwini participants (Table 1).

**Table 2:** Participant reports of reimbursement expenditure: quantitative data

What reimbursement money is spent on^a^	eThekwini % (n)	Umgungundlovu % (n)	Total % (n)
Transport to research site	86.4 (216)	75.6 (189)	81.0 (405)
Toiletries	62.0 (155)	66.0 (165)	64.0 (320)
Basic foods/groceries	30.4 (76)	44.0 (110)	37.2 (186)
Take away foods	11.6 (29)	40.8 (102)	26.2 (131)
Childcare during study visits	15.2 (38)	17.6 (44)	16.4 (82)
Fashion clothes	7.2 (18)	21.2 (53)	14.2 (71)
Hair care	5.6 (14)	16.4 (41)	11.0 (55)
Special make-up/skin care	6.0 (15)	8.4 (21)	7.2 (36)
Nail care/treatments	2.0 (5)	3.2 (8)	2.6 (13)
Airtime/data	2.0 (5)	0.4 (1)	1.2 (6)
Transport to school/work	1.2 (3)	1.2 (3)	1.2 (6)
Rent	0.4 (1)	1.6 (4)	1.0 (5)
Electricity	0.8 (2)	0 (0)	0.4 (2)
Other (diapers, underwear, wine)	0 (0)	1.6 (4)	0.8 (4)

^a^Multiple response options given

Similarly, in the FGDs, participants reported that they spent the majority of their reimbursement money on transport. After transport, the most common expenditures reported by FGD participants were toiletries and essential foodstuffs. Participants’ descriptions of toiletries included personal hygiene products like toothpaste, sanitary pads, or deodorant, and some also listed cosmetic items such as perfumes, body lotions or make-up products as purchases.

*It also helps me to buy cosmetics. Yah, and have good things. (Participant 8, eThekwini, FGD 2)*

One of the Umgungundlovu participants spoke of her concern for personal hygiene during the study visits as motivating her need to buy appropriate toiletries:

*[S]ometimes we have to come smelling nicely and clean because it won’t be right to come with bad odour while counselors are helping us, you see. We are just not alright. Odours. It helps us to also buy, you buy cosmetics, be beautiful and bath, and be able to bath. (Participant 3, Umgungundlovu, FGD 1)*

During the FGDs, the majority of participants from both research sites reported that they used any additional reimbursement money according to what they lacked at the time they received it. Many women described that the reimbursement money supplemented their income, and how they used any extra money to cover their basic costs of living. Some women made decisions about what to do with any additional reimbursement money according to their financial situation at the time: if they needed food at home they bought it, but if they had sufficient basic essentials when they received their reimbursement they might spend the money on clothing, hair treatments or takeaway food.

*…it depends on whether my [study visit] date came when the month was still wet [meaning when someone just got paid and they still have money to spend] or when it is about to end. If my date was about to end [end of month] I pass by and pick up isishebo [meat, poultry, vegetables, stews, curries, etc served with starchy foods] in the house. I then travel. But if […] I do not have my hair plaited, I am able to relax [her hairstyle] or maybe buy a skirt. (Participant 6, eThekwini, FGD 2)*

*It helps me where I lack at that time […] Sometimes you find that I am short of transport fare to go to work. Sometimes you find that I top up the child’s lunch items. Maybe I buy what is short in the house. (Participant 7, Umgungundlovu, FGD 1)*

*It’s not the same. It depends on how I am at that time. Most of the times it coincides with the time when I am lacking in the house in terms of food. (Participant 6, Umgungundlovu, FGD 1)*

Some participants enjoyed the financial independence the reimbursement money gave them, especially since many relied on financial support from others.

*This money helps me because I… if I am short of something I am able to buy it myself, without having to always ask from my mother. (Participant 2, eThekwini, FGD 2)*

In both the quantitative and qualitative data, expenditure on non-essentials such as takeaways or hair treatments were reported. Some reimbursement money was also sporadically spent on rent, debt repayments, clothing lay-byes, school books, and cell phone credit.

*[M]ost of the youth is at home and is not working. So, this money helps in every way it can help. Sometimes you get to find that, okay, you find that you didn’t know what you would eat but since you know that okay at a certain date… you are able to borrow money. Then when you have gotten the reimbursement money, you go and patch up where you borrowed from. (Participant 3, Umgungundlovu, FGD 1)*

One woman used the money to further her employment search.

*It helped me before when I wanted to submit CVs. There is no one who can just give you money and say “Go and submit CVs”. Firstly, you have to go to the internet cafés with the CV and do it. This money helped me to go and do the CV, to also be able to apply for a job. So, it helps a lot (Participant 3, Umgungundlovu, FGD 1)*

### Ideal Reimbursement Amount

**Fig. 1:**
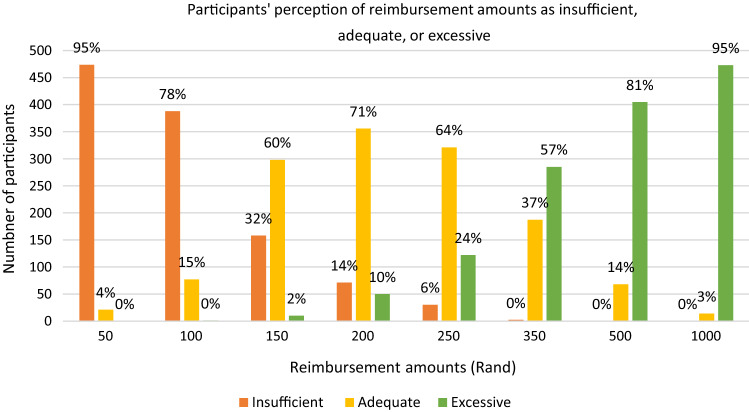
Participants’ perception of adequacy of reimbursement amounts

Participants who completed the quantitative questionnaire were asked what amount of reimbursement they would consider “insufficient, adequate or excessive” (Fig. 1). What participants considered as adequate reimbursement per study visit was largely between ZAR150 (US$10) and ZAR350 (~ US$24).

Similarly, during the FGDs, most participants described that ZAR300 (~ US$20) was an ideal amount for a study visit. ZAR150 (~ US$10) (the amount paid to them per visit—at enrolment, month one, month three, and thereafter three monthly follow-up visit intervals—for participation in the study) was deemed by many in the FGDs to be too little.

*[I]t’s [ZAR150] too little because you come here once after two months, right? [participant error, visits were every three months, except for first check-up visit which was after 2 months] So, you have been broke for a very long time. At least [if we were given] R300… [laughter] in this R300 you can be able to budget and really do something significant, as this sister is saying that maybe you can buy isishebo [curry/stew] on the way because those are the things which quickly run out (Participant 5, eThekwini, FGD 1)*

However, a few participants were satisfied with the amount (ZAR150, ~ US$10) paid to them. Some acknowledged other benefits from participation, such as altruism and healthcare services. These women felt the reimbursement was an additional bonus, even if it was not much.

*P01: [I]n my opinion, I don’t have a problem with R150, in fact, whatever amount because in fact joining this study, it’s not because we have come to hold the pick-axe [to work hard] […] There is someone who ends the day without the R150. […] In fact, we have come to help by joining this study, so that this research is done and to achieve what they want at the end. So, it is about helping others’ lives. […] I don’t have a problem with whatever amount it is because we are not at work. (Participant 1, Umgungundlovu, FGD 1)*

*[W]hat we get is okay. We benefit here and there. And the help, to know about your health here, we benefit. We also thank the staff here who are hospitable to us, who attend to us really well. But I would be happy if R150 would be added and make it R300. (Participant 7, eThekwini, FGD 2)*

One woman recognised that, for herself and those like her, the reimbursement amount would never be enough to satisfy them because their needs were too great.

*We’ve got too much needs. If we can say reimbursement must satisfy all our needs, maybe we need R20000 [*~*US$1 379] each for coming here. So, reimbursement will never satisfy our needs […] Life is too expensive than the reimbursement money (Participant 2, Umgungundlovu, FGD 2)*

In the FGDs, participants stated that they believed the reimbursement amount should be according to type of procedure undertaken but were divided amongst themselves regarding whether or not payment schedules should increase over time in recognition of their commitment to the study or remain stable because procedures were the same at every visit.

*[M]oney should be the same because same things are done, right. Blood draws are done for checking, STIs are checked. So, it has to be the same because the same thing is done. (Participant 1, eThekwini Clinic, FGD1)*

*I think that it would be alright if it can increase at the next stage, maybe to encourage, to encourage us […] just acknowledging the fact that the person is still in the study, she has not disturbed the study. (Participant 1, Umgungundlovu, FGD1)*

*The fixed one [amount] is alright […] Because if it keeps increasing, it’s like we are at work. (Participant 3, Umgungundlovu, FGD1)*

## Discussion

Although most participants used some of the reimbursement money to cover their transport costs to the research centre, many also used it to supplement the costs of everyday existence and survival, such as basic groceries or to buy small luxuries, such as toiletries. Many women in the study were unemployed and relied on social grants for their income. The prioritisation of expenditure was often dependent on how participants perceived their economic situation to be when they received their reimbursement payment. There were only minor differences in spending habits between the sites. Participants from Umgungundlovu District spent slightly less on transport than eThekwini District participants, which is possibly related to geographic ease of access—more participants from Umgungundlovu District lived closer to the research site. As they paid less overall in transport costs, it is unsurprising that Umgungundlovu participants spent more than eThekwini District participants on almost every other type of expenditure.

Many participants used reimbursement money to purchase toiletries and personal care products. When discussing personal care expenditure, they mentioned financial autonomy, the desire to treat themselves, wanting to smell nice (including in preparation for study visits), or simply that they were able to buy things that they had run out of. The additional reimbursement was sometimes regarded as a ‘bonus’ which allowed some women to treat themselves occasionally if their circumstances allowed. As the majority of the women were young and not economically independent, the desire to buy items related to physical appearance and presentation may be a powerful motivating factor in these types of expenditures. Some of the reimbursement was used to purchase food—basic groceries and takeaways. Participants purchased food to supplement what they had at home, often referring to provisions for their children. Similar, gendered spending habits have been noted in South African adolescents enrolled in a cash transfer program [29], where females focused their spending on domestic support and personal care items.

In order to be able to assess an appropriate reimbursement amount that does not impact on ability to consider risk without being exploitative, participants were asked to rate reimbursement value. Previous literature [16] has shown that when reimbursement is perceived as insufficient, it negatively impacts participant accrual, retention and morale—and this can happen during the course of a study when cost of living increases. Some FGD participants referred to rising costs as a reason to increase reimbursement amounts. The updated SAHPRA guidelines (2018) suggest a minimum payment of ZAR300 (~ US$20) at present which therefore acknowledges inflation—and this amount was deemed acceptable to participants in our study [7]. Furthermore, this amount is less than amounts provided by government social grants which ranged from approximately R400 (~ US$27.50) for child support to R1690 (~ US$116.50) for old age pension at the time of the study [28]. Similar to other studies, some participants had enrolled for various perceived study related benefits including health monitoring and education, accessing quality healthcare, and altruism [1, 30, 31]. These participants deemed any amount of reimbursement as an added bonus.

As in any research, there are limitations to this study. The data presented represent a small sub-sample population within a larger study. Furthermore, the sampled population were from only two geographic areas and therefore generalisability is difficult to infer within a wider context. However, the qualitative data imparted by the participants provides depth and details to substantiate the quantitative data and is instrumental in conveying general themes common to the lives of many South African women. Finally, as the monetary worth of reimbursement payments to participants is linked to the volatile local inflation rate, the implicit value is inherently unstable and therefore difficult to quantify.

## Conclusions

According to our data, the updated SAHPRA guidelines (2018) recommending reimbursement of ZAR300 (~ US$20) per standard visit are consistent with participants’ perceptions of appropriate reimbursement [7]. Reimbursement, as well as access to quality healthcare, are powerful motivators for participation in trials in low-resource settings [30, 31], and this highlights the importance of well-designed studies and sound informed consent processes in order to protect participants [22] as well as data integrity [15]. During times of economic recession, reimbursement through trial participation will be even more attractive to vulnerable groups, and the need to verify true voluntariness of consent becomes even greater. Ascertaining a fair transaction between researchers and participants should go beyond reimbursement amounts to ensuring participants are exposed to minimal risk, and are able to make informed, empowered and entirely voluntary choices.

## Data Availability

Access to the data from this ancillary study of the ECHO Study may be requested through submission of a research concept to: cmilford@mru.ac.za. The concept must include the research question, data requested, analytic methods, and steps taken to ensure ethical use of the data. Access will be granted if the concept is evaluated to have scientific merit and if sufficient data protections are in place. As of the time of publication, data access applications are in process with the governing institutional review boards of the ECHO Study to make de-identified data from the primary ECHO dataset publicly available.

## Footnotes

1 At an exchange rate of approximately US$1 to ZAR14.5 (February 2021).
